# WebMGA: a customizable web server for fast metagenomic sequence analysis

**DOI:** 10.1186/1471-2164-12-444

**Published:** 2011-09-07

**Authors:** Sitao Wu, Zhengwei Zhu, Liming Fu, Beifang Niu, Weizhong Li

**Affiliations:** 1 Center for Research in Biological Systems, University of California San Diego, La Jolla, California 92093, USA

## Abstract

**Background:**

The new field of metagenomics studies microorganism communities by culture-independent sequencing. With the advances in next-generation sequencing techniques, researchers are facing tremendous challenges in metagenomic data analysis due to huge quantity and high complexity of sequence data. Analyzing large datasets is extremely time-consuming; also metagenomic annotation involves a wide range of computational tools, which are difficult to be installed and maintained by common users. The tools provided by the few available web servers are also limited and have various constraints such as login requirement, long waiting time, inability to configure pipelines etc.

**Results:**

We developed WebMGA, a customizable web server for fast metagenomic analysis. WebMGA includes over 20 commonly used tools such as ORF calling, sequence clustering, quality control of raw reads, removal of sequencing artifacts and contaminations, taxonomic analysis, functional annotation etc. WebMGA provides users with rapid metagenomic data analysis using fast and effective tools, which have been implemented to run in parallel on our local computer cluster. Users can access WebMGA through web browsers or programming scripts to perform individual analysis or to configure and run customized pipelines. WebMGA is freely available at http://weizhongli-lab.org/metagenomic-analysis.

**Conclusions:**

WebMGA offers to researchers many fast and unique tools and great flexibility for complex metagenomic data analysis.

## Background

Metagenomics is a new field that studies the environmental microorganism populations using culture-independent sequencing technologies. It provides revolutionary and unprecedented view of the identities, dynamics and functions of microbial communities in various environments such as marine [1], human gut [2] and many others [3-5].

The recent advances in next-generation sequencing technologies [6] such as 454, Illumina, SOLiD and HeliScope significantly promoted the development of metagenomics by offering low-cost and ultra-high throughput sequencing. Huge amounts of available metagenomic sequence data create tremendous challenges in data analysis. Some challenges are computational and result from the huge quantity of sequence data. It can easily consume 10^4~5 ^CPU hours to query a regular 454 sample with 10^6 ^reads against NCBI's non-redundant (NR) database using BLAST [7]. Other challenges are due to the high complexity of metagenomic sequence data: (a) a sample may contain hundreds or thousands of species at dramatically different abundance levels; (b) many species are unknown; (c) next-generation sequencers produce shorter reads with higher error rate compared to Sanger sequencers; and (d) sequence data contain other experimental bias, artifacts and contaminations [8]. To address these problems, many methods have been developed such as taxonomy binning [9-11], use of simulated datasets [12], diversity analysis [13], ORF calling [14,15], rRNA prediction [16], sequence clustering [17-20], assembly [21], statistical comparison [22], fragment recruitment [1,8,23] and so on. For example, Megan [11] assigns taxonomic groups to query sequences based on BLAST search against a reference database, usually the NCBI NR. CD-HIT has been used in clustering raw reads and ORFs to identify non-redundant sequences or gene families [24]. Mothur [25] is a software package with several functions such as identification of Operational Taxonomic Units (OTUs). QIIME [26] is another useful package for the investigation of microbial diversity using rRNAs. Software package RAMMCAP [27] provides a very fast sequence clustering and annotation pipeline.

It is very difficult for common researchers to install and maintain so many software tools needed in metagenome annotation. Many users simply do not have the required computational resources to run some of the tools. The available online resources that provide metagenomic data analysis are also limited. Currently, MG-RAST [28] and CAMERA [29] are the major sites where users can submit datasets for analysis. MG-RAST only provides a fixed pipeline and the waiting time for its jobs is often very long (sometimes weeks). CAMERA offers a list of workflows, but many useful tools are still missing from CAMERA's site. In addition, both MG-RAST and CAMERA require user registration and login, so it is difficult to access their web servers using scripts.

In order to provide a fast, easy and flexible solution for metagenomic data analysis, we developed WebMGA, a web server that allows users to submit metagenomic datasets and to run many kinds of analysis, or to perform a user-customized annotation pipeline. WebMGA is freely available at http://weizhongli-lab.org/metagenomic-analysis to all users without any login requirement.

### Implementation

**Figure 1:**
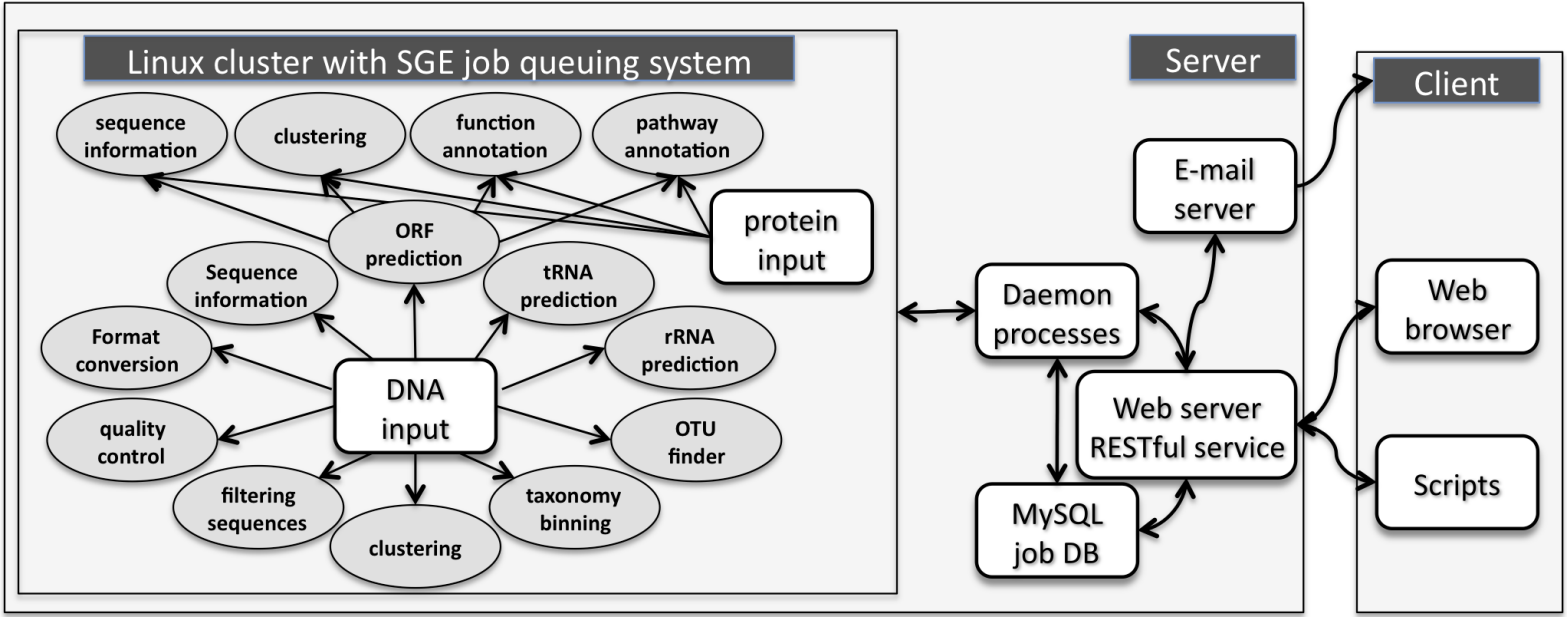
**Illustration of WebMGA and its metagenomic analysis functions**. The major input of WebMGA is either a DNA sequence file or a protein sequence file. A user can run a single analysis at a time such as to prediction ORFs from the uploaded DNA sequences. A user can also use a script to call WebMGA to run multiple analyses or run a pipeline where one job can use the output of another job.

WebMGA consists of a web user interface, web service interface, server scripts, a MySQL relational database, an email server, daemon processes, application software packages, wrapping and parsing scripts and a computer cluster (Figure 1). The WebMGA web front-end is an Apache HTTP server, which accepts jobs submitted through web browsers. WebMGA's web services, which are implemented with Mojolicious software, accept client-side scripts following Representational State Transfer (REST) protocol. Job requests are processed by server scripts, which submit jobs to a queue and return a unique job identifier with a web link for each request. If an email address is provided (optional), the user will be notified by email of job status change. All the job-related data such as job identifiers, status, date and time are stored in the MySQL database, and managed by server scripts and daemon processes. The daemon processes handle the job queue, submit jobs to computer cluster and check job status. A user can query the status or retrieve the results of a job, using web browser or scripts, by submitting a job identifier. The latest versions of software packages are locally installed on our computer cluster, which runs Linux operating system and Sun Grid Engine job queuing system. We implemented scripts to run these applications in parallel and parse the outputs.

## Results and Discussion

### Computational tools

As outlined in Figure 1, WebMGA includes a wide range of tools for analyzing large and complex metagenomic sequence datasets. WebMGA is implemented with many tested tools that can process millions of sequences in minutes to hours. The key features of WebMGA are: (a) rapid analysis enabled by very fast algorithms and methods, (b) a large collection of computational tools, (c) flexibility to run individual tools or configure a pipeline consisting of individual tools, and (d) compatibility of application and pipelines with both web browsers and client-side scripts.

WebMGA currently has 26 individual tools that cover the following categories:

•**Quality control **has 3 tools to filter or trim raw reads and yield high quality reads. The first tool (QC-filter-FASTQ) takes reads in FASTQ format and yields high quality reads in FASTA format. The second tool (QC-filter-FASTA-qual) takes a FASTA file and a quality score file and generates high quality reads in FASTA format. The third tool (Trim) trims low-quality tails of inputted Illumina reads using SolexaQA [30].

•**Sequence clustering **has 4 tools: CD-HIT-EST, CD-HIT, H-CD-HIT [17-20] and CD-HIT-454 [31]. The first two take DNA and protein sequences as input respectively, perform clustering, and output clusters and non-redundant sequences. H-CD-HIT is a 2-step clustering analysis for proteins. The program first performs clustering on the input dataset and the representatives of this step are the input of the second clustering round. H-CD-HIT produces a hierarchical structure for proteins; it also maximizes the computational efficiency and the quality of clustering. CD-HIT-454 takes raw 454 reads and identifies the artificial duplicates, which are commonly present in 454 pyrosequencing reads.

•**rRNA identification **includes BLASTN-rRNA [16] and HMM-rRNA [16]. BLASTN-rRNA identifies rRNA from DNA fragments by querying against 5S Ribosomal Database, European ribosomal RNA database and SILVA database [32-34] through BLAST. Despite BLASTN-rRNA shows higher specificity than HMM-rRNA for 5S rRNA prediction, HMM-rRNA, an HMM-based method, has much higher speed and overall better sensitivity. For more detailed comparison between these two tools, please refer to reference [16]. Both programs take DNAs in FASTA format and output 3 files: predicted rRNA sequences in FASTA format, a 'TAB' delimited text file that lists the rRNA type and positions, and a FASTA file for the original input sequences with the predicted rRNAs masked by letter 'N'. The purpose of the masked file is to prevent false ORF calling if it is used for ORF prediction.

•**tRNA identification **uses tRNA-scan [35] to identify tRNAs from the inputted DNA sequences. Similar to rRNA tools, it outputs 3 files: the predicted tRNA sequences, a 'TAB' delimited text file, and a masked input file.

•**ORF calling **include 3 tools for ORF prediction from DNA sequences: ORF-finder [27], Metagene [14] and FragGeneScan [15]. ORF-finder calls ORFs by translating all six reading frames, where an ORF starts at the beginning of a sequence or the first ATG after a previous stop codon and ends at the first stop codon or the end of that sequence. ORF-finder covers more true ORFs and yields more spurious ORFs than Metagene and FragGeneScan. It is more suitable to use when the inputted DNA sequences are below 200 bp. Metagene is the first *ab initio *ORF prediction program that is designed for fragmented sequences. FragGeneScan is another *ab initio *program that can handle frame-shift errors, which are typical for 454 reads. All these three tools take nucleotide sequences as input and output ORF protein sequences in FASTA format.

**Figure 2:**
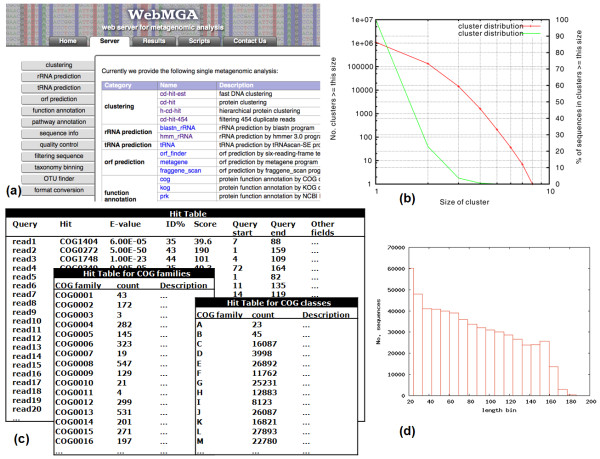
**A screenshot and examples of output results of WebMGA**. (a) A screenshot of WebMGA server (b) A plot of distribution of clusters by CD-HIT (c) COG annotation results are in several "TAB" delimited text files, which can be easily viewed locally. (d) A plot of length distribution by sequence statistical tool

•**Function annotation **includes 5 tools. We implemented scripts to annotate the inputted peptide sequences against PFAM and TIGRFAM families using HMMER3 [36] and against NCBI's COG, KOG and PRK databases using RPS-BLAST. We output the annotation in 'TAB' delimited text files, which include the details of hits of each query against each reference family (alignment position, e-value, score etc) and also several derived results. For example, for COG annotation, we also give summarized results of number of hits to each COG family and each class (Figure 2c). For PFAM search, we also provide Gene Ontology (GO) annotation through the mapping between FPAM and GO.

•**Pathway annotation **takes peptide sequences in FASTA format as input, searches our curated KEGG database with BLASTP, and generates the pathway annotation in 'TAB' delimited text files. The reference KEGG database was prepared to speed up the BLASTP search. We clustered the KEGG database at 90% sequence identity, and if the sequences in one cluster all belong to the same KO group, only the representative sequence (the longest one) of this cluster is used in the reference database. Otherwise (rare situation), all sequences in that cluster are used. Compared to the original KEGG database, searching the curated database recovers > 99% of the hits and is ~10 times faster.

•**Sequence statistics **has 2 tools: FNA-stat and FAA-stat. They take nucleotide (FNA-stat) or protein sequences (FAA-STSAT) as input and output the summary information of the inputted file including length distribution, GC content etc (Figure 2d).

•**Filtering human sequence **is a filtering tool for identification of human sequences from human microbiome samples. This tool queries the inputted reads against human genome and mRNAs using FR-HIT [8]. If the similarity between a read and a human sequence meets a user-specified cutoff (e.g. 95% identity over 90% of the read length), this read is filtered out. FR-HIT can identify similar number of hits as BLASTN, but it is about 2 orders of magnitude times faster than BLASTN. This tool produces a file of un-filtered reads in FASTA format and a text file that lists the filtered reads along with alignment information to human reference sequences.

•**Taxonomy binning **has 2 tools: RDP-binning and FR-HIT-binning. The first uses the binning tool in Ribonsomal Database Project (RDP) [37] to bin rRNA sequences. The second tool aligns the inputted metagenomic reads to NCBI's Refseq database and then assigns the reads to the taxon that is the Lowest Common Ancestor (LCA) of the hits. LCA was originally introduced in Megan [11], where BLAST is used for alignment. Since BLAST is too slow for large metagenomic datasets, FR-HIT is utilized here.

•**OTU clustering **takes rRNA tags and clusters them into OTUs. The software we used here is CD-HIT-OTU (to be described in a separate publication), which is a clustering program we developed that can process millions of rRNAs in a few minutes, while some traditional methods such as MOTHUR [25] and ESPRIT [38] need days for millions of sequences. CD-HIT-OTU is also more accurate than many traditional methods that tend to overestimate the diversity due to sequence errors.

•**File conversion **is a tool that converts reads from FASTQ format to FASTA format.

### Individual web servers

Each of the 26 tools introduced above was implemented as a standalone web server. As illustrated in the screenshot of WebMGA web server (Figure 2a), each tool has its own web page so that users can upload DNA or protein sequences for analysis, e.g. to call ORFs from raw reads using FragGeneScan. Different applications generate different type of files including sequence files in FASTA or FASTQ format (e.g. ORF or RNA prediction), TAB delimited text files (e.g. COG annotation, Figure 2c), graphic files (Figure 2b, d), raw output files and so on. Due to the great diversity of the output types, particular visualization pages are not available for all tools. The results produced by WebMGA and documentation are packed into a zip file for a user to download and analyze at client-side.

### Interactively perform analysis pipelines

**Figure 3:**
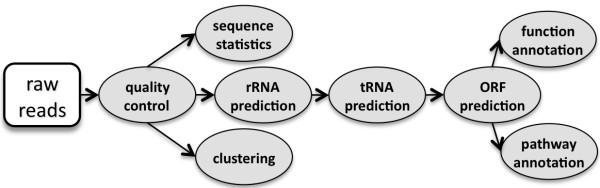
**A simple example pipeline configured with tools in WebMGA**.

Most metagenomic data analysis pipelines include many processes using different tools. Figure 3 gives a simplified pipeline as an example. With WebMGA, users can run complex pipelines by interactively using the individual web servers. For example, to run the pipeline in Figure 3, a user can upload the raw reads to the quality control tool and then input the high-quality reads into "sequence statistics", "rRNA prediction" and "clustering" servers and run them in parallel. Once the rRNA prediction is completed, the user can download the result and use the masked sequences (one of the output files from rRNA prediction) as input to run tRNA prediction followed by ORF-finder. When ORF-finder is finished, function and pathway annotation jobs can be submitted in parallel using the predicted ORFs as input.

### Client-side scripting

One advantage of interactively running a pipeline is that a user can monitor and control the annotation process, for example, by checking the results and choosing suitable programs and parameters in the next step. But this way may be too tedious for routine analyses. WebMGA offers RESTful web services for all the tools through which a complex pipeline can be automatically executed using one client-side script. Two template Perl scripts, client_submit_job.pl and Rammcap_client_submit.pl, are available at WebMGA web site. A user can straightforwardly use the template Perl scripts to configure an annotation pipeline and run it locally.

The first template script runs a single tool: it submits DNA sequences in a FASTA file to CD-HIT-EST web service and downloads the clustering results. The second script performs a more extensive annotation using RAMMCAP pipeline [27], which is also used by CAMERA project. This script starts with a FASTA file of reads and then runs a list of web services such as sequence statistics, clustering, rRNA and tRNA finding, ORF calling, and function annotation and finally downloads all the annotation results.

### Computational time and throughput

Three datasets were used to test the performance of tools in WebMGA. The first one is a metagenomic sample (ID F3T1Le1) selected from a core gut microbiome study [39], which contains 555,853 reads with an average length of 252 bps. The second dataset contains 571,261 ORFs with an average length of 66 letters predicted from the first dataset using Metagene [14] with default parameters. The third dataset, which contains 33 16S rRNA samples from study [39], has 817,942 16S rRNA reads spanning the V6 variable region (average length 78 bps).

The wall time and total CPU time for each tool to process the above datasets are listed in Table 1. Fast tools like sequence statistics, file conversion, quality control, rRNA-scan and ORF calling use only one CPU core; clustering tools use 4 cores in parallel; other relatively time-consuming jobs use up to 40 cores. When our cluster has enough free cores for WebMGA, about 50% and 75% of jobs can complete within 10 minutes and 1 hour respectively. All jobs need less than 3 hours except the slowest pathway annotation against KEGG, which needs about 20 hours.

**Table 1:** Computational time and throughput for each tool of WebMGA

Category	Tool	Data^a^	Wall time (h:m:s)	Total CPU time (h:m:s)	Daily throughput^b^
Clustering	CD-HIT-EST	1	00:08:53	00:34:08	3,113

	CD-HIT	2	00:00:58	00:02:52	23,040

	H-CD-HIT	2	00:20:06	01:10:26	1,600

	CD-HIT-454	1	00:05:40	00:21:54	4,800

rRNA	BLASTN-rRNA	1	00:12:43	13:44:53	139

	hmm-rRNA	1	00:01:56	00:20:35	5,008

tRNA	tRNA-scan	1	00:02:29	02:01:50	936

ORF calling	ORF-finder	1	00:02:06	00:02:06	23,040

	Metagene	1	00:16:21	00:15:21	6,400

	FragGeneScan	1	01:27:50	01:27:50	1,294

Function	COG	2	00:14:55	15:12:50	126

	KOG	2	00:15:16	16:25:31	116

	PRK	2	00:28:38	32:03:16	59

	PFAM	2	01:33:44	115:30:23	16

	TIGRFAM	2	00:53:23	62:31:51	30

Pathway	KEGG	2	20:24:33	553:32:48	3

Statistics	FNA-stat	1	00:00:38	00:00:38	43,746

	FAA-stat	2	00:00:12	00:00:12	52,363

Quality control	QC-filter-FASTQ	1	00:03:13	00:03:13	19,200

	QC-filter-FASTA-qual	1	00:02:47	00:02:47	23,040

	Trim	1	00:04:00	00:04:00	16,457

Filtering	Filter-human	1	00:40:28	02:29:57	762

Binning	RDP-binning	1	01:16:30	01:20:00	1,404

	FR-HIT-binning	1	00:36:59	02:13:53	853

OTU clustering	CD-HIT-OTU	3	00:05:10	00:10:23	8,861

File conversion	FASTQ2FASTA	1	00:02:24	00:02:24	23,040

^a ^See text for descriptions of the 3 datasets tested.

^b ^Daily throughput is calculated as the daily CPU time of WebMGA cluster with 80 cores divided by the total CPU time of a job, assuming 2 minutes of administrative CPU cost such as job queuing, file coping etc. for each job.

We allocated 80 CPU cores from our cluster for WebMGA server to use exclusively. With this computational capacity, WebMGA can process hundreds of jobs with most tools per day (Table 1). For example, the daily throughput for ORF-finder is about 23,000 based on the second dataset. Function and pathway annotations are the bottlenecks, but WebMGA can still process 3 (i.e. KEGG) to more than one hundred datasets (e.g. COG) in a day. WebMGA only allows 1 KEGG job to run with up to 40 cores at the same time so that other fast jobs can be completed quickly.

### Example

To illustrate the application of WebMGA, we annotated the first test dataset (i.e. the core gut microbiome sample F3T1Le1) using the template script Rammcap_client_submit.pl. Since this dataset was already filtered by the original authors, we skipped the quality control, duplicates clustering and FILTER-HUMAN steps. The annotation summaries are outlined in Table 2. The results are comparable to those published in the reference [39]. For example, the relative abundance of COG categories annotated in this example shows no visible difference to that in original literature (Supplementary Figure 17b) [39].

**Table 2:** Annotation summary for example dataset

Tool	Annotation Summary^a^
FNA-stat	Total reads: 555853, Length: 45~607, Average length: 251, Total bases: 139813458, Total ambiguous bases: 96190, Distribution of GC% and length in text files and in graphic files similar to Figure 2d

CD-HIT-EST	Parameters: "-d 0 -n 10 -l 11 -r 1 -p 1 -g 1 -G 0 -c 0.95 -aS 0.8" Clusters: 419802, Size of the largest cluster: 69, Clusters in CD-HIT format and in 'TAB' delimited text file, Distribution of clusters in graphic file similar to Figure 2b

HMM-rRNA	rRNA sequences identified: 3858, Archaeal-16S: 1, Eukaryotic-18S: 3, Bacterial-16S: 1347, Bacterial-5S: 220, Bacterial-23S: 2285, Archaeal-5S: 2

tRNA-SCAN	tRNA sequences identified: 1378

Metagene	ORFs: 571261

FAA-stat	Total ORFs: 555853, Length: 20-121, Average length: 66, Total letters: 37859696, Total ambiguous letters: 87294, Distribution length in text file and in graphic file similar to Figure 2d

CD-HIT	Parameters: "-d 0 -n 5 -p 1 -g 1 -G 0 -c 0.9 -aS 0.8" Clusters: 396559, Size of the largest cluster: 154, Clusters in CD-HIT format and in 'TAB' delimited text file, Distribution of clusters in graphic file similar to Figure 2b

COG	Parameters: "-e 0.001" Total alignments: 199002, Total ORFs aligned: 198933, Total COG families aligned: 2848, Total COG classes aligned: 23, Alignments and derived results in 'TAB' delimited text files similar to Figure 2c

PFAM	Parameters: "-e 0.001" Total alignments: 187156, Total ORFs aligned: 174115, Total PFAM families aligned: 3131, Total ORFs with GO annotation: 123294, Total GO terms annotated: 964 Total ORFs with EC annotation: 46207, Total EC terms annotated: 319 Alignments and derived results in 'TAB' delimited text files similar to Figure 2c

TIGRFAM	Parameters: "-e 0.001" Total alignments: 6357, Total ORFs aligned: 6172, Total PFAM families aligned: 327, Total ORFs with GO annotation: 3077, Total GO terms annotated: 252 Total ORFs with EC annotation: 564, Total EC terms annotated: 57 Alignments and derived results in 'TAB' delimited text files similar to Figure 2c

^a ^Detailed parameters are explained at WebMGA website.

### Comparison to other web servers

In metagenomics, MG-RAST and CAMERA are the dominating web servers that provide online data analysis. Both resources have been constantly busy and many jobs submitted to them need to wait long time for completion. For example, we also submitted gut sample F3T1Le1 to both MG-RAST and CAMERA for annotation and it took them 5 days and 12 hours respectively. WebMGA used 4.5 hours to annotate the same dataset using RAMMCAP pipeline. WebMGA adds additional computational resources for the increasing need in metagenomic data analysis.

Compared with both MG-RAST and CAMERA, the most important advantage of WebMGA is the flexibility to run user-customized analysis pipelines with client scripts besides web server interface. MG-RAST has a fixed annotation pipeline that users cannot modify, which is essential to compare annotations of different samples. However a fixed pipeline is not suitable for all the diverse requirements in metagenomic studies, where researchers need to use different tools and different parameters. CAMERA has many analysis workflows that can process user-uploaded data. But these tools can only be used interactively by users that are logged in.

MG-RAST and WebMGA share many common procedures such as quality control, filtering and clustering, but they also apply different methods or resources for the same type of annotations. Here are some examples: (1) MG-RAST treats the reads whose first 50 bases are identical as duplicates, but WebMGA uses CD-HIT-454 for this purpose. MG-RAST's method is faster but may miss the duplicates with sequence errors (indels and wrong base calls) within the first 50 bases. CD-HIT-454 is slightly slower, but is more sensitive and can pick the duplicates missed by MG-RAST. (2) For host associated samples, MG-RAST uses bowtie [40] to identify near identical matches to host reference sequences and removes these reads as host contaminations. WebMGA uses a slower but more sensitive method, FR-HIT, for human-contamination removal. (3) For ORF calling, MG-RAST uses FragGeneScan; while WebMGA allows users to choose from ORF-finder, Metagene and FragGeneScan.

CAMERA and WebMGA also have many common methods, mostly because CAMERA also adopted the RAMMCAP pipeline we developed. But WebMGA has many unique tools such as Filter-HUMAN, RDP-binning, FR-HIT-binning and CD-HIT-OTU that CAMERA doesn't have.

## Conclusions

In order to assist researchers in the metagenomics field to deal with data analysis challenges, we implemented WebMGA with very fast algorithms and effective methods. With WebMGA, users can use many individual tools and assemble the tools into a pipeline for more complicated analysis through web browsers or client-side scripts. We are in the process of developing new tools and validating more public tools so that, in the future, more rapid tools and pipelines will be added to WebMGA server.

### Availability and requirements

•**Project name**: WebMGA

•**Project home page: **http://weizhongli-lab.org/metagenomic-analysis

•**Operating system(s): **Platform independent

•**Programming language: **Perl (client-side scripts)

•**Other requirements: **browsers

•**License: **no license needed

•**Any restrictions to use by non-academics: **no restriction

## Authors' contributions

SW, ZZ and WL contributed to system concept. SW and ZZ implemented the system and performed major programming work. LM and BL contributed to the development of CD-HIT software and FR-HIT software, respectively. SW, ZZ and WL coordinated this work, contributed the data analysis and wrote the manuscript. All authors read and approved the final manuscript.
